# Nutritional assessment of plant-based beverages in comparison to bovine milk

**DOI:** 10.3389/fnut.2022.957486

**Published:** 2022-08-08

**Authors:** Nick W. Smith, Anant C. Dave, Jeremy P. Hill, Warren C. McNabb

**Affiliations:** ^1^Sustainable Nutrition Initiative, Riddet Institute, Massey University, Palmerston North, New Zealand; ^2^Fonterra Research and Development Centre, Palmerston North, New Zealand

**Keywords:** plant-based milk alternatives, compositional analysis, dietary choice, alternative proteins, sustainable diets

## Abstract

Plant-based beverages (PBB) are often marketed and used by consumers as alternatives to ruminant milks, particularly bovine milk (hereafter referred to as milk). However, much research has established that there is variation in nutritional composition among these products, as well as demonstrating that they are largely not nutritional replacements for milk. A survey of the prices and nutrition labels of PBB available in New Zealand supermarkets was undertaken. Selected almond, coconut, oat, rice, and soy PBB products were then analyzed for nutritional content, including energy, fat, protein, amino acid, bioavailable amino acid, and trace element contents. Finally, the protein and calcium contents of well-mixed and unshaken products were analyzed to ascertain the impact of colloidal stability on nutrient content. All PBB groups were more expensive than milk on average, while their declared nutrient contents on package labels was highly variable within and between groups. Analyses of selected PBB revealed that soy products had the most similar proximate composition to milk, while all other PBB groups contained less than 1.1 g protein per 100 mL on average. Many PBB were fortified with calcium to a similar concentration as that in milk. Shaken and unshaken samples showed divergent protein and calcium content for several PBB products but had no effect on the composition of milk, indicating that the nutrient content of PBB at the point of consumption will be dependent on whether the product has been shaken. Only the soy PBB had comparable amino acid content and bioavailability to milk. Overall, our results demonstrate the diversity in composition and nutritional properties of PBB available in New Zealand. While the existent environmental footprint data on PBB shows that they generally have lower carbon emissions than milk, milk currently accounts for approximately 1% of the average New Zealand resident’s consumption-based emissions. Except for calcium-fortified soy PBB, none of the commercially available PBB had nutritional compositions that were broadly comparable to milk.

## Introduction

Plant-based beverages (PBB) are a product category experiencing increasing consumer demand and diversity of offering. PBB defy a single definition due to the variety of their forms but are often marketed as alternatives to bovine milk (hereafter referred to just as milk) and attempt to replicate the functionality and sensory properties of milk.

Milk remains a key part of the diet for much of the global population. In total, 62% of United States households consumed dairy and little or no PBB in 2019 (1). Of the remainder, 16% of households regularly purchased both milk and PBB, and 23% purchased almost exclusively PBB. Globally, dairy is an important contributor to population nutrition, providing 49% of global food calcium, 15% of dietary fat, and 12% of dietary protein (2). Evidence is also clear in the literature for the beneficial health impacts of milk consumption across a variety of outcomes (3), whereas the health impacts of PBB are less clear, likely given their diversity and more recent emergence as widely consumed items (4, 5).

PBB are gaining popularity, with sales in the United Kingdom increasing 32% from 2019 to 2020, taking the market value of PBB to around 12% the size of milk (6). PBB are often chosen by consumers for: dietary and health reasons, including as a dairy substitute for those with lactose intolerance and dairy allergies; as a lower fat option; for inclusion in a vegan diet; due to beliefs on animal welfare; or due to perceived reductions in environmental footprints (7).

Here, the price and nutritional value of PBB on the New Zealand market were analyzed. Both product label data and nutritional content derived from laboratory analysis were employed, due to previous results highlighting lower nutrient contents in PBB than stated on product labels (8).

## Materials and methods

### Compilation of nutritional panel information

PBB information was collected from supermarket stores owned by the two major New Zealand supermarket chains and from their websites, between January and June 2021. The stores visited were in Palmerston North, New Zealand. The collected information included price, pack size, serving size and nutritional panel information. Some additional nutritional information was collected from product manufacturer websites.

The survey was restricted to ambient shelf-stable PBB products; products requiring chilled storage were not selected for comparison. Similarly, PBB products declared as protein shakes or smoothies were excluded from comparisons in this manuscript. Three fresh chilled and three ambient stable ultra-heat treated (UHT) milk products were surveyed for comparison, all of which were marketed as “standard” milk, in terms of fat content.

The PBB were grouped according to their type (e.g., soy, almond, etc.). The information collected from each type was averaged for data presentation. In total, 103 PBB products from 28 brands were available from these retailers and were surveyed.

### Sample analyses

Due to the high number of PBB products on the New Zealand market, only a subset could feasibly be analyzed for nutritional content. A selection of at least three products for each PBB product type and for the milk products was chosen. Selection was based on similar caloric contents to the type average, and cost (covering high-, median-, and low-cost products for each type). Product types where fewer than three individual products could be sourced (e.g., hemp and cashew) were included in the nutritional panel survey, but omitted from any further analysis. As stated above, fresh chilled milk and ambient stable UHT milk were treated as separate product types with the same selection criteria as used for PBB. See Supplementary material for details of the products selected.

Each product was mixed thoroughly by shaking vigorously at least 10 times while in the original packaging. In total, 500 mL of the product was then sampled into coded containers. The samples were mixed with sodium azide [0.02% w/w; Llopis et al. (9)] as a preservative and the containers were sealed. The sealed containers were sent to an external accredited laboratory (Hill Laboratories, Hamilton, New Zealand) for experimental analysis. The samples were analyzed for macronutrient (fat, protein, total carbohydrate including sugars profile, and dietary fiber), mineral, and amino acid content. The analytical values obtained for samples for each parameter were averaged within the product type for comparison.

PBB are known to undergo settling during storage. To understand the impact of this colloidal instability on the nutritional composition, selected products were subjected to undisturbed storage for 7 days at 4°C. This technique was employed to simulate the behavior of products during storage by consumers. After this storage duration, a 250 mL aliquot of the sample from the top of the package was carefully removed using a pipette, without shaking the samples. The aliquot from each of the products was put in coded containers, mixed with sodium azide (0.02% w/w), and sent for proximate analysis to an external accredited laboratory.

Total nitrogen content was measured using Dumas combustion (AOAC 992.15, 19th edition). Gross energy was measured using a bomb calorimeter (AC500 Leco Corporation, United States). Total dietary fiber content was measured using a Megazyme kit (AOAC 991.43). Content of simple sugars was measured using gas liquid chromatography with flame ionization detection. Acid stable amino acid contents were measured *via* HCl hydrolysis followed by reversed phase high-performance liquid chromatography (HPLC) using AccQ Tag derivatization (AOAC 994.12). Tryptophan content was measured *via* alkaline hydrolysis, and cysteine/methionine *via* performic acid oxidation (AOAC 994.12). Reactive lysine content was measured *via* guanidination with o-methylisourea and HPLC (10). The remaining nutrient contents measurements were performed *via* biological materials digestion, nitric and hydrochloric acid micro-digestion and filtration, and analysis by inductively coupled plasma optical emission spectroscopy (ICP-OES).

The protein content of the samples was derived from the analyzed nitrogen content. To calculate the true protein contents of the products, specific nitrogen conversion factors for each product type were used. The following values were obtained from the literature for use here: milk, 6.38; almond, 5.18; oat, 5.83; rice, 5.95; and soy, 5.71 (11–13). For coconut proteins, no specific nitrogen conversion factor was found and hence the standard conversion factor of 6.25 was used.

### Data analysis

Descriptive statistics were calculated for product types. Where multiple prices existed for the same product, the cheapest was used in the data analysis.

New Zealand dollars (NZD) were used for all cost calculations throughout the analysis. Recommended daily nutrient intake values for a 60 kg 30-year-old woman were used throughout, obtained from national guideline documentation (14) or international sources (15). The New Zealand guidelines did not feature quantitative recommendations for sugar, so one was taken from guidelines for the United Kingdom (16).

## Results

### Packaging information and price analysis

Table 1 details the price and proximate compositions compiled from the nutritional panels of different PBB and milks available in New Zealand supermarkets. The cost of fresh milk ranged from $2.46 to $2.98 per liter (mean $2.80) and UHT ranged from $1.90 to $3.39 (mean $2.60). The retail price of the PBB varied between types (mean of $3.93 per liter of soy product to mean of $6.29 for hemp product) but also within types ($10.07 difference between the cheapest and most expensive almond products). All PBB types were more expensive than milk on average, but several individual almond, soy, and rice products were cheaper than the most expensive milks surveyed.

**TABLE 1:** Collated retail price and nutritional label data for all products sampled.

	Milk (fresh) *N* = 3	Milk (UHT) *N* = 3	Almond *N* = 37	Cashew *N* = 2	Coconut *N* = 13	Hemp *N* = 2	Oat *N* = 12	Rice *N* = 7	Soy *N* = 30
Price per liter (NZD)	2.80 (2.46–2.98)	2.60 (1.90–3.39)	4.94 (2.79–12.86)	3.99 (3.99–3.99)	5.32 (3.79–8.48)	6.29 (6.29–6.29)	4.69 (2.99–6.98)	4.30 (2.50–6.40)	3.93 (2.49–7.98)
% of title ingredient**	NA	NA	4 (2–10)	3*	25 (4–59)	4*	13 (9–16)	20 (13–40)	9 (4–17)
**Content per 100 mL**									
Energy (kJ)	265 (263–270)	270 (260–280)	130 (67–275)	98 (73–123)	186 (95–354)	146 (108–184)	263 (193–612)	224 (209–256)	221 (126–305)
(Cal)	63 (63–65)	64 (62–67)	29 (16–50)	24 (18–29)	29 (23–33)	36 (26–45)	55 (48–71)	53 (51–55)	53 (32–65)
Protein (g)	3.5 (3.3–3.9)	3.6 (3.5–3.9)	0.8 (0–3.5)	0.5 (0.4–0.5)	0.7 (0.2–1.5)	0.4 (0.3–0.5)	1.1 (0.2–2.3)	0.4 (0–0.6)	3.2 (1.9–4.2)
Fat, total (g)	3.4 (3.3–3.4)	3.5 (3.4–3.5)	1.9 (1.1–3.8)	1.5 (1.4–1.5)	3.3 (1.4–7.9)	2.7 (2.7–2.7)	2.1 (1–3.1)	1.1 (0.5–1.3)	2.4 (1.3–3.8)
Carbohydrate (g)	4.8 (4.7–4.8)	4.7 (4.7–4.8)	2.5 (0–6.5)	2.2 (0.8–3.6)	3.1 (0.3–7.8)	2.4 (0.1–4.6)	7.8 (6–11.7)	10.4 (9.5–12)	4.4 (0.4–8.9)
Sugars (g)	4.8 (4.7–4.8)	4.7 (4.7–4.8)	1.8 (0–6.3)	1.5 (0.1–2.8)	2.3 (0.2–5.9)	2.3 (0–4.6)	3.2 (1–4.5)	5.7 (3.1–10.6)	2.6 (0.4–7.1)
Dietary fiber (g)	ND	ND	0.4 (0.2–1.9)	0.1 (0.1–0.1)	0.3 (0–0.9)	0 (0–0)	0.9 (0.3–2.4)	0.4 (0–1)	0.6 (0–1.8)
Sodium (mg)	38 (35–40)	42 (40–45)	45 (21–108)	41 (40–42)	24 (12–61)	0 (0–0)	42 (32–52.6)	44 (6–68)	51 (16–93.5)
Potassium (mg)	ND	ND	37 (18–127)	19 (18–19)	64 (0–150)	ND	237 (237–237)	ND	200 (143–260)
Calcium (mg)	123 (117–135)	127 (122–135)	101 (7–128)	120 (120–120)	93 (16–120)	ND	99 (3–120)	108 (80–120)	108 (10–160)
Phosphorus (mg)	ND	ND	76 (74–82)	58 (56–59)	46*	ND	100 (100–100)	ND	102 (60–122)

Data displayed as: mean (range). Note that this table includes data from flavored and formulated PBB products. Data for further nutrients available in Supplementary material.*Data was only available from a single product.**Title ingredient is the nut, cereal, or crop from which the product was made, e.g., almonds for almond products, soybeans for soy product, etc. ND, no data.

The composition of milk showed minimal variation between products. The composition of the PBB varied between and within product types. The caloric contents of the PBB ranged from 73 kJ/100 mL (cashew product) to 1034 kJ/100 mL (coconut product). Most PBB had added sugar in their ingredient declarations, with the exceptions of the oat and rice products (Supplementary material). An analysis of the nutrients contributing to the calories suggested that most of the calories resulted from the fat and/or carbohydrates (added sugars) in PBB. Most PBB were fortified with calcium salts and had approximately 100 mg calcium per 100 mL declared in the ingredient list and nutritional panel on their labels; however, there were products of every PBB type that had no calcium content. Listed contents in the PBB that were not listed for milk included dietary fiber, potassium, and phosphorus, although milk was later shown to contain potassium and phosphorus at levels within or exceeding the range shown by the PBB (Table 2).

**TABLE 2:** Analyzed contents of selected samples for each product type.

	Milk (fresh) *N* = 3	Milk (UHT) *N* = 3	Almond *N* = 3	Coconut *N* = 4	Oat *N* = 4	Rice *N* = 3	Soy *N* = 3
Total solids (g/100 g)	11.77 ± 0.21	12.03 ± 0.15	3.1 ± 0.3	5.57 ± 1.52	10.73 ± 0.88	10.9 ± 1	10.45 ± 1.34
Gross energy (kJ/100 g)	278 ± 3	284 ± 4	84 ± 4	128 ± 9	217 ± 19	189 ± 8	256 ± 31
Total dietary fiber (g/100 g)	NA	NA	0.36 ± 0.11	0.45 ± 0.18	0.85 ± 0.8	0.09 ± 0.03	0.72 ± 0.46
Total fat (g/100 g)	3.3 ± 0.1	3.3 ± 0.1	1.6 ± 0.1	2.1 ± 0.51	1.9 ± 0.8	1 ± 0.17	2.7 ± 0.1
Protein (g/100 g)	3.34 ± 0.31	3.62 ± 0.16	0.78 ± 0.12	0.78 ± 0.65	0.8 ± 0.22	0.4 ± 0.07	2.87 ± 0.07
**Sugars**							
Fructose (g/100 g)	<0.1	<0.1	<0.1	<0.5	<0.1	<0.1	<0.1
Glucose (g/100 g)	<0.1	<0.1	<0.1	<0.1	1.23 ± 2.4	1.63 ± 2.31	<0.1
Lactose anhydrous (g/100 g)	4.27 ± 0.06	4.1 ± 0.1	<0.1	<0.1	<0.2	<0.1	<0.1
Lactose monohydrate (g/100 g)	4.47 ± 0.06	4.3 ± 0.1	<0.1	<0.1	<0.2	<0.1	<0.1
Maltose (g/100 g)	<0.1	<0.1	<0.1	<0.1	1.55 ± 1.04	2.9 ± 1.73	<0.1
Sucrose (g/100 g)	<0.1	<0.1	0.1 ± 0	1.63 ± 1.27	0.1 ± 0	0.1 ± 0.06	1.73 ± 0.32
Galactose (g/100 g)	<0.1	<0.1	<0.1	<0.1	<0.1	<0.1	<0.1
Total sugar (g/100 g)	4.27 ± 0.06	4.1 ± 0.1	<0.5	1.83 ± 1.42	2.93 ± 2.03	4.57 ± 0.64	1.8 ± 0.26
Calcium (g/100 g)	0.11 ± 0.01	0.12 ± 0.005	0.09 ± 0.06	0.1 ± 0.03	0.07 ± 0.05	0.12 ± 0.03	0.1 ± 0.07
Magnesium (g/100 g)	0.01 ± 0.0003	0.01 ± 0.0002	0.01 ± 0.003	0.006 ± 0.002	0.004 ± 0.0005	0.004 ± 0.001	0.013 ± 0.002
Potassium (g/100 g)	0.16 ± 0.001	0.16 ± 0.005	0.02 ± 0.0009	0.08 ± 0.11	0.13 ± 0.11	0.02 ± 0.01	0.2 ± 0.05
Sodium (mg/100 g)	48.67 ± 0.58	48 ± 1.73	57.33 ± 1.53	41 ± 16.02	61.5 ± 7.85	71.33 ± 14.19	67 ± 24.25
Phosphorus (g/100 g)	0.09 ± 0.004	0.1 ± 0.004	0.05 ± 0.03	0.07 ± 0.03	0.07 ± 0.05	0.04 ± 0.02	0.09 ± 0.04
Iron (mg/100 g)	<0.05	<0.05	0.1 ± 0.03	0.15 ± 0.01	0.09 ± 0.01	< 0.05	0.38 ± 0.13
Copper (mg/100 g)	<0.005	<0.005	0.02 ± 0.002	0.03 ± 0.014	0.022 ± 0.012	0.008 ± 0.002	0.059 ± 0.027
Iodine (mg/100 g)	0.0032 ± 0.0009	0.005 ± 0.0026	0.0065 ± 0.0004	0.0011 ± 0.0006	0.0016 ± 0.0033	<0.0005	0.0083 ± 0.0004
Selenium (mg/100 g)	<0.01	<0.01	<0.001	<0.001	<0.001	<0.001	<0.001
Zinc (mg/100 g)	0.32 ± 0.04	0.35 ± 0.02	0.07 ± 0.01	0.06 ± 0.04	0.04 ± 0.02	0.05 ± 0.02	0.14 ± 0.07

Values shown are mean ± SD across the samples analyzed for each product type.

Several benefit claims were made on product labels. These included: less processed; permeate free; and calcium content for the milk products. For the PBB, these included: fiber content (up to 2.4 g per 100 mL); cholesterol free; low or zero lactose content; high in unsaturated fats; low in saturated fat; unsweetened; and no added sugar.

The protein content of the milk products was 3.3–3.9 g per 100 g. This was similar to the protein content in the majority of soy products. The other PBB product types had on average less than 1 g per 100 g protein. The soy products had the highest protein content of the PBB [mean 3.21 g per 100 g (range 1.9–4.2)], while the rice products had the lowest protein content [mean 0.38 g per 100 g (range 0–0.6)]. The almond PBB showed the widest variation in protein content (0–3.5 g per 100 g). Combining price and protein data, the mean cost per gram of protein was >$0.60 for all PBB except soy (mean $0.12). The highest protein cost of any product was $3.00 per gram, for one of the oat products. In comparison, the mean cost per g of protein in the milk products was $0.08.

**FIGURE 1:**
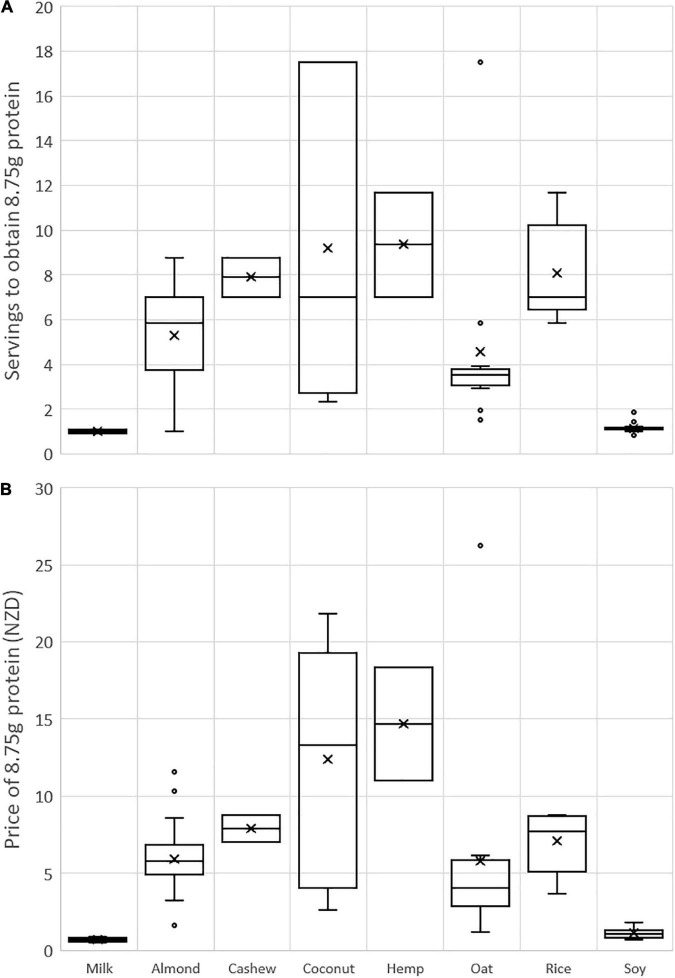
Comparison of the number of servings **(A)** and the price **(B)** to obtain 8.75 g protein (equivalent to one serving of milk) from PBB. A serving size of 250 g was assumed for all products. Products with no protein content were omitted. The × symbol denotes the mean value; boxes show the median and interquartile range; range bars show the minimum and maximum values, excluding outliers (circles) that are more than 1.5 times the interquartile range below or above the first or third quartile, respectively.

Figure 1 shows the number of servings and the price of achieving the same amount of protein (8.75 g) as a 250 g serving of milk for each PBB group. Coconut products showed the greatest variation in both servings and price. The soy products had similar protein costs to milk.

### Analyzed nutrient content

Table 2 shows the aggregated analyzed nutrient content values by product type. The milk products had the highest energy content at around 280 kJ per 100 g, while the almond products had the lowest at around 80 kJ per 100 g. The mean dietary fiber contents of the PBB ranged from 0.09 to 0.85 g per 100 g. The milk products had the highest fat content (3.3 ± 0.1 g per 100 g), while the rice products had the lowest (1 ± 0.17 g per 100 g). For the sugars, the oat and rice products were the only two with measurable glucose and maltose content, while the milk products were the only products with measurable lactose. All PBB had measurable sucrose content; the milk products did not. The highest total sugar content was in the rice products (4.57 ± 0.64 g per 100 g), while the almond products had concentrations below 0.5 g/100 g.

Mean calcium content was relatively constant across the samples (0.07–0.12 g per 100 g). However, the variation in calcium content was comparatively greater for the PBB than for milk. The milk products had the highest phosphorus content. The soy products had the highest copper, iodine, iron, magnesium, potassium, and zinc contents. The rice products had the highest sodium content. All products had only trace selenium concentrations.

**FIGURE 2:**
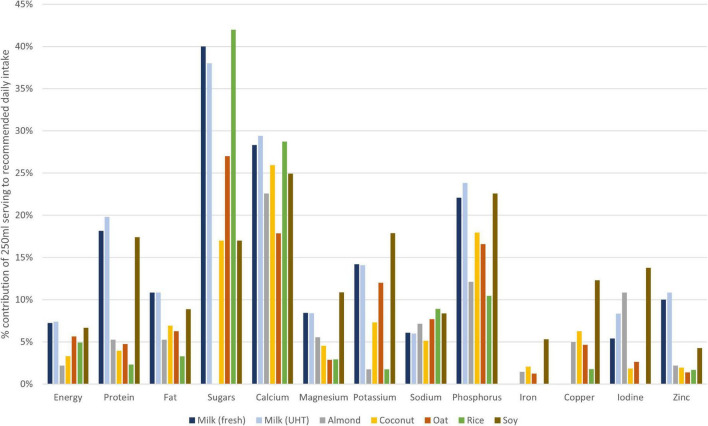
Contribution of a single 250 mL serving of each product type to an adult woman’s recommended daily intake for selected nutrients. Bars show the mean nutrient content across the analyzed samples for each product type.

Figure 2 compares the contribution of a serving of each product type to recommended daily intake (RDI) of the nutrients analyzed. For most nutrients, the milk or soy products contributed the greatest percentage of RDI. The exceptions were: sugars and sodium, for which a serving of the rice products made the greatest contribution; iron and copper (negligible content in milk); iodine (highest contributions from soy and almond PBB); and calcium, to which all product types contributed 18–29% of RDI.

Table 3 displays the protein cost analysis for the different product types. Due to the lower mean price and higher protein content of milk, the price per g protein for these products was the least of all the product types. The soy products had the lowest cost per g protein of the PBB types, while the rice products had the highest.

**TABLE 3:** Analysis of protein price for each product group.

	Milk (fresh)	Milk (UHT)	Almond	Coconut	Oat	Rice	Soy
NZD to meet protein RDI from product	3.77	3.24	28.36	35.77	26.47	48.78	6.15
NZD/g protein	0.08	0.07	0.63	0.79	0.59	1.08	0.13

The mean price of the analyzed products for each product type was used.

**FIGURE 3:**
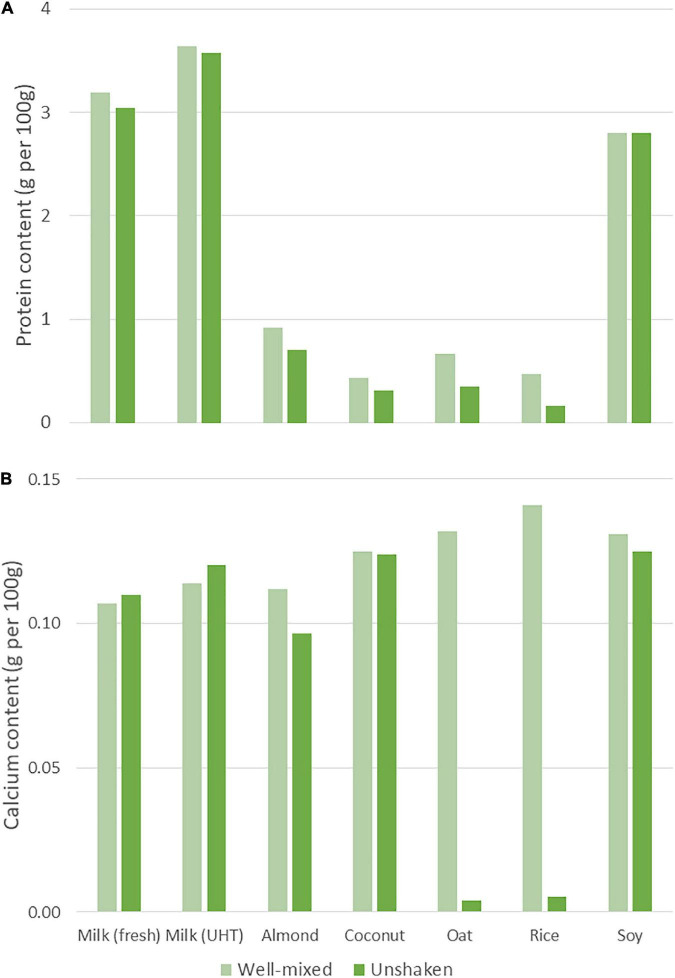
Comparison between the protein **(A)** and calcium **(B)** content of well-mixed samples and unshaken samples. A single representative product was analyzed for each product type.

To understand the impact of colloidal instability on the nutritional composition of samples, a single product from each type was selected and samples from the product with and without shaking were compared (Figure 3).

The protein content of the unshaken milk and soy product samples deviated by a maximum of 5% from the well-mixed samples. In comparison, the protein content of the other unshaken PBB samples were 24–66% lower than the well-mixed samples. For calcium, the content of the unshaken samples for milk and coconut PBB deviated by a maximum of 6% from the well-mixed samples. The unshaken almond and soy samples had 14 and 18% lower calcium contents than their well-mixed samples, whereas the unshaken rice and oat samples had 96 and 97% lower calcium contents than the well-mixed samples, respectively.

Finally, the amino acid content of each product type was analyzed. As shown in Table 4, a 250 mL serving of the milk products would provide the greatest contribution toward the RDI of all amino acids considered (29–61% contribution to each amino acid), except for histidine, for which soy PBB provided an equal contribution to fresh milk. For the other PBB, the contribution to amino acid intakes was 11% or less.

**TABLE 4:** Mean percentage of an adult woman’s recommended daily intakes for amino acids supplied by one 250 ml serving of each product type.

	Cow’s milk (fresh)	Cow’s milk (UHT)	Almond	Coconut	Oat	Rice	Soy
Histidine	29%	33%	6%	5%	5%	2%	29%
Isoleucine	33%	36%	5%	5%	5%	1%	27%
Leucine	32%	34%	5%	4%	5%	1%	24%
Lysine	36%	38%	2%	4%	3%	1%	25%
SAA	33%	37%	4%	5%	9%	4%	23%
AAA	54%	61%	10%	8%	11%	5%	44%
Threonine	38%	42%	5%	5%	6%	2%	31%
Tryptophan	41%	43%	6%	8%	9%	0%	37%
Valine	34%	40%	4%	5%	6%	2%	23%
Reactive lysine (as a % of total lysine)	95 ± 2	94 ± 2	73 ± 3	69 ± 13	49 ± 9	40 ± 21	99 ± 1
Reactive lysine (as a % of lysine recommended daily intake)*	34%	36%	1%	3%	1%	<1%	25%

SAA, sulfur amino acids (methionine and cysteine); AAA, aromatic amino acids (phenylalanine and tyrosine). Amino acid requirements obtained from FAO (15) and calculated for 60 kg adult. *Calculated by multiplying the % lysine supplied by one serving by the mean reactive lysine %.

As a proxy for amino acid bioavailability, the reactive lysine content of each product was also quantified for each analyzed product [Table 4; Rutherfurd et al. (17)]. Lysine was chosen as a focus as it is often the most limiting indispensable amino acid in the human diet (17, 18). In total, 99% of soy PBB lysine was reactive, and the values for the milk products were above 90%. The rice products had the lowest reactive lysine proportion at a mean of 40%. However, these percentages should be seen in the context of total lysine content: while the soy PBB had the highest reactive lysine percentage, the higher lysine content of milk lead to the greatest absolute reactive lysine content being found in milk.

## Discussion

Plant-based beverages are widely consumed as substitutes in foods and meals that traditionally contained milk. However, the recently emerged and growing body of literature comparing the nutritional properties of PBB to milk has demonstrated that they are not nutritionally equivalent (19–25). This study, for the first time, quantifies the nutritional differences between milk and the PBB available on the New Zealand market.

The nutritional information reported on product labels varied, with some reporting content for an extensive list of nutrients, while others reported minimal information. There was considerable variation in the reported composition of the PBB. Compared to the PBB, from both product labels and the analyzed samples, the milk products assessed were cheaper and had a higher protein, energy, fat, sugar, potassium, phosphorus, and zinc content than the PBB. Contrastingly, the PBB had higher fiber, sodium, iron, and copper contents than milk. The PBB type most comparable to milk in terms of cost and nutrient content was the soy type. This was true for several nutrients, but notably protein and the amino acids. Protein and calcium were focused on in this report due to the importance of milk to the global supply of these essential nutrients in food (2) and their important role in the current New Zealand diet (26, 27).

In this study, PBB products were more expensive both per unit volume and per gram of protein than milk (Table 3). Indeed, except for the soy products, the majority of PBB products had protein contents below 1 g per 100 g. This may be attributed to the lack of a robust regulatory framework for PBB in New Zealand. While milk must contain at least 3 g protein per 100 g, among other requirements (28), there are currently no regulations in New Zealand controlling the nutritional content of PBB. This indicates the need for a review of regulatory standards for PBB to better control their nutritional content if they are to be marketed as alternatives to milk.

Previously, Drewnowski et al. (29) proposed standards for PBB nutritional content, particularly those marketed as “milks.” These standards encompassed energy, protein, sugar, saturated fat, sodium, calcium, and vitamin content, as well as protein quality and optional guidelines for fiber, carbohydrates, and potassium. The standards reflected the nutritional quality of milk, with additional plant-specific nutrient standards based on soy PBB. These authors surveyed 641 existing PBB products on the US market and found that <5% (all of which were soy products) met their criteria, largely due to low protein content.

Applying their standards to the product labels surveyed in this study, it was found that a maximum of 22 out of 103 PBB products (21%) would qualify. This set of 22 contained 20 soy products, one almond product, and one rice product. However, not all products reported values for every assessed nutrient on their labels and assessment of protein quality was beyond the scope of this study. Thus, it is likely that fewer of these 22 products would meet the strictest standards proposed. Drewnowski et al. (29) also proposed that 1–2 g of fiber per 100 g was a suitable target for PBB; only nine of the 103 products surveyed here stated fiber contents above 1 g per 100 g. New Zealand regulations require that only products with a fiber content above 2 g per serving may make nutrition content claims relating to dietary fiber, which is a stronger condition than proposed by Drewnowski et al. (29) and would exclude all but one of the surveyed products (30). The United States results complement those presented here and the authors questioned whether the majority of PBB available to consumers should be marketed as “milks.”

It has been reported elsewhere that 90% of PBB in the USDA Branded Food Products Database met the criteria for ultra-processed foods due to the presence of added caloric or non-caloric sweeteners, hydrogenated oils, hydrolyzed proteins, flavors, flavor enhancers, emulsifiers, emulsifying salts, thickeners, and bulking and gelling agents, added salt, and/or added fat (31). These additives were also found in the product survey carried out here (Supplementary material). The author concluded that “dietary guidelines that promote plant-based diets but penalize industrial processing may need to acknowledge the fact that most PBB milk alternatives… are ultra-processed foods.”

Many other authors have performed analyses of the nutritional content of PBB and made similar conclusions to those presented here. The majority of these studies analyzed product labels or label databases alone, finding similarly high variability in the nutritional content of different products as reported here, both between and within PBB types (19–25). Others have undertaken laboratory analysis of the nutritional content of PBB, again with conclusions matching those reported here (8, 25, 32–34).

Previous work has also considered the glycemic index (GI) of PBB in comparison to milk, finding that rice and coconut PBB had the highest GI, and milk the lowest (32). Others considered price, finding that PBB products (as well as plant-based alternatives to yogurt and cheese) on the United Kingdom market were all more expensive than milk, in some cases up to twice the price (22). By matching their nutritional analysis to United Kingdom dairy intakes, these authors identified cost and nutritional risks of substituting milk with PBB, particularly to consumer groups for whom milk makes a major contribution to nutrient intakes and/or those who have higher nutrient requirements, such as children and pregnant women.

Interestingly, single-serve PBB products have been found to have higher nutritional content than multi-serve products (such as those analyzed here). An analysis of 51 such products on the US market found that these products generally had higher protein content, and higher levels of calcium and vitamin B12 fortification (35). This was likely due to the raised nutrient requirements of the target consumers of the products: children and the elderly. However, only 18% of these products met the nutritional requirements for inclusion in United States school lunch programs.

Several studies on the nutritional content of PBB have emphasized that, while their content of nutrients commonly associated with milk are low, these products contain other essential nutrients not found in milk. Iron, copper, and fiber are often mentioned, as well as plant-specific compounds, such as plant sterols (33, 36). The contribution of a serving of PBB to the RDI for these essential nutrients was found to be small here, with soy products making the greatest contributions to iron (5%), copper (12%), and iodine (14%) RDI. The remaining PBB made lesser contributions, and as mentioned earlier, the fiber content of most PBB was below 1 g per 100 g.

The low nutrient content of PBB is partly due to their low content of the named plant ingredient. Using the values in Table 1, a 250 g serving of the almond products surveyed here contained on average 9 g of almonds, which is around 6 almonds. For the other PBB, a 250 g serving contains on average 22 g soy, 33 g oats, 50 g rice, and 63 g of coconut. The low nutrient content is thus unsurprising given the low content of nutritious plant material. Further, the ability of the human digestive system to absorb these nutrients from PBB must be considered.

It has been found that the bioaccessibility of phosphorus and zinc is significantly lower in soy PBB products compared to milk (34). The bioavailability of other nutrients, particularly calcium, is also a concern. The calcium contents of the majority of surveyed and analyzed products were within 30% of the content of milk. However, for the PBB products this was largely the result of fortification (see ingredient lists in Supplementary material). Phytate, an antinutrient found in plant-based foods and beverages, reduces mineral absorption from plant-based foods, motivating advice to fortify and combine these with animal-sourced foods and vitamin C-rich foods in situations where nutrition is a priority (37).

The results presented here also caution assuming that nutrients are equally distributed throughout PBB containers. While the impact of shaking product containers was only analyzed for calcium and protein, the trends suggest that similar results would be found for other nutrients. The observed reduced nutrient content of unshaken products matches previous results for calcium. Eight soy PBB products tested for their calcium content, were found on average to have 31% of the calcium content stated of the label when unshaken, due to sedimentation (8). Upon shaking, this increased to 59% of the label stated value, with the remaining calcium content found in solid residue at the bottom of the containers. Our own results described here identified not only discrepancies between the nutritional content of shaken versus unshaken PBB products, but also between the nutritional contents described on their labels versus the content found upon laboratory analysis of shaken samples (Supplementary material).

The discussion of bioavailability must also extend to protein quality. The Digestible Indispensable Amino Acid Score (DIAAS) captures the digestibility of the indispensable amino acids in a food, as well as their ratio in comparison to human requirements (15). DIAAS scores above 1 are considered excellent sources of protein, able to meet the complete amino acid requirements of the consumer, while scores between 0.75 and 1 are good sources of protein, and scores below 0.75 can make no nutritional claim on protein quality. Milk protein concentrate has a DIAAS score of 1.18, compared to 0.94–0.97 for soy protein isolate, 0.54 for cooked rolled oats, 0.4 for almonds, and 0.37 for rice protein concentrate (38, 39). No value was found for coconut. Thus, most PBB types can make no nutritional claim on protein quality, except soy, which is a good source of protein. Moreover, the soy product analyzed here was the only PBB type with a reactive lysine proportion similar to (and exceeding) that of milk. However, it should be noted that this is only a proxy measure for amino acid bioavailability, and that after adjustment for reactivity, the lysine content of the soy PBB remained lower than milk.

Previous work has recommended a blending of legume and grain proteins in PBB to achieve higher overall protein quality (22, 25, 29). While the combination of complementary proteins will address some of the deficits of indispensable amino acids in the products containing only one protein source, the total protein content of products should also be considered.

Some consumers choose PBB over milk for perceived reductions in the environmental impacts of their diets. Indeed, it has been found that milk has roughly twice the carbon footprint of soy PBB by mass on the Italian market (40). In a broad review of life cycle analyses, milk had on average three times the greenhouse gas emissions (CO_2_-equivalents), more than ten times the land use, and higher terrestrial acidification, eutrophication, and scarcity-weighted freshwater withdrawals than soy PBB per liter (41). Life cycle analysis data for other PBB is rare and highly variable, due to their more recent emergence as product categories and their smaller production volumes. However, recent reports suggest GHG emissions between 35% less (oat) and 70% more (rice) than soy PBB per serve (25). Consistent global data on other footprints was only found for soy.

It is important to understand the context of these footprints. Food and non-alcoholic beverages accounted for 24% of New Zealand household consumption-based emissions in 2019 (42). Using data from two analyses of the climate impact of the New Zealand diet, we calculated that New Zealand milk consumption (approximately 200 g per person per day) accounted for around 4.7% (0.3 kg CO_2_-equivalents) of an individual’s emissions attributable to diet (43, 44). Combining these percentages, removal of milk from the diet would reduce an individual’s total consumption-based emissions by around 1.1%. This is without considering the emissions related to foods added to the diet to replace milk. For example, using the mean nutrient content values presented here and carbon footprints from Singh-Povel et al. (25), if one were to replace the protein content of milk with an almond, coconut, or rice PBB, this would result in a net increase in emissions. This reflects the results of Singh-Povel et al. (25), who showed that meeting nutritional requirements for amino acids with milk incurred GHG emissions of 312 g CO_2_-equivalents, with higher emissions for all other PBB except soy (160 g CO_2_-equivalents).

While the GHG emissions from milk production are significantly higher than those of PBB per serve, milk’s high nutrient density relative to soy and oat PBB resulted in equal scores on a combined nutrition and climate impact metric (45). Elsewhere, using a recently developed Nutrient-Rich Food Index combined with an Environmental Impact metric, milk outperformed an unfortified oat beverage twofold, but a calcium-fortified oat beverage outperformed milk (46). The combination of life cycle analysis data with nutritional indices is a nascent field without defined standards for calculation, so such scores must be interpreted with caution (47, 48). However, it is essential when considering the environmental impact of two food products that their respective nutritional value and function in the diet also be taken into account.

## Conclusion

Given the results presented here and previously by other authors, soy PBB appear the nearest nutritional substitute for milk, with several caveats pertaining to sedimentation, calcium bioavailability, protein quality, and price. While individual consumers will consider price, nutritional value, and environmental impact differently or not at all in purchasing decisions, it is important that the scientific and regulatory community understand the implications of milk and PBB consumption.

This research adds to the international consensus that PBB should in general not be considered as nutritional substitutes for milk (21, 22). Indeed, recommendations exist from physicians’ societies that PBB and milk are not interchangeable, particularly in the diets of infants and young children, due to the reduced nutritional value of PBB (49).

While PBB can certainly be of dietary value to those unable (due to health constraints) or unwilling to consume milk, the nutritional contribution of these products should be more widely understood to ensure consumption of nutrient adequate diets. This is particularly relevant for children and pregnant women. In the future, regulations that drive production of PBB products with nutritional contents meeting proposed standards would help to achieve this goal.

## Data availability statement

The original contributions presented in this study are included in the article/Supplementary material, further enquiries can be directed to the corresponding author.

## Author contributions

JH and WM conceived the idea for the work. AD oversaw the sample collection and analysis. NS and AD analyzed the data and wrote the manuscript. All authors contributed to review of the manuscript and approved its submission for publication.
